# Neurotensin and Adverse Cardiovascular Outcomes in Patients Undergoing Percutaneous Coronary Intervention

**DOI:** 10.3389/fcvm.2022.782602

**Published:** 2022-03-08

**Authors:** Maximilian Tscharre, Serdar Farhan, Matthias K. Freynhofer, Michael Leutner, Sabina Baumgartner-Parzer, Ioannis Tentzeris, Birgit Vogel, Florian Tinhofer, Miklos Rohla, Thomas W. Weiss, Kurt Huber, Alexandra Kautzky-Willer

**Affiliations:** ^1^Department of Internal Medicine, Cardiology and Nephrology, Landesklinikum Wiener Neustadt, Wiener Neustadt, Austria; ^2^Institute of Cardiometabolic Diseases, Karl Landsteiner Society, St. Pölten, Austria; ^3^The Zena and Michael A. Wiener Cardiovascular Institute, Icahn School of Medicine at Mount Sinai, New York, NY, United States; ^4^Medical Department, Cardiology and Intensive Care Medicine, Hospital Ottakring, Vienna, Austria; ^5^Department of Internal Medicine III, Clinical Division of Endocrinology and Metabolism, Unit of Gender Medicine, Medical University of Vienna, Vienna, Austria; ^6^Medical School, Sigmund-Freud University, Vienna, Austria; ^7^Gender Institute, Lapura Women's Health Resort, Gars am Kamp, Austria

**Keywords:** neurotensin, acute coronary syndromes, stable coronary artery disease, percutaneous coronary intervention, adverse outcomes

## Abstract

**Background:**

Neurotensin is involved in fatty acid and glucose metabolism and promotes the development of obesity and diabetes. These associations appear to be more pronounced in women. We investigated the association of neurotensin with long-term major adverse cardiovascular events (MACE) in patients presenting with acute coronary syndrome (ACS) or chronic coronary syndrome (CCS) undergoing percutaneous coronary intervention (PCI).

**Methods:**

We included 452 consecutive patients [144 (31.9%) females] undergoing PCI for ACS or CCS. Plasma samples drawn after PCI were analyzed for neurotensin with an enzyme-linked immunoassay. As primary endpoint, a composite of MACE including all-cause death, non-fatal myocardial infarction and non-fatal stroke during 7 years of follow-up was investigated. As secondary endpoint, we investigated all-cause death.

**Results:**

Neurotensin levels did not differ between male and female patients (*p* = 0.560). MACE occurred in 150 (33.2%) patients. Restricted cubic splines demonstrated a U-shaped association of log-transformed neurotensin with the primary and secondary endpoint. Therefore, we dichotomized our cohort according to tertiles of log-transformed neurotensin. In Kaplan-Meier analysis including the total cohort and restricted to male patients log- neurotensin tertiles were not associated with MACE (both *p* > 0.05). Moreover, in the overall cohort and in male patients multivariable Cox regression analysis log-neurotensin tertiles were not associated with MACE or with all-cause death (all *p* > 0.05). However, in female patients log-neurotensin was associated with MACE in Kaplan-Meier analysis (log-rank *p* = 0.013). Also, after multivariable adjustment female patients in the first tertile had a significantly increased risk for MACE compared to female patients in the second tertile [HR 3.84 (95% CI 1.71–8.60), *p* = 0.001]. There was tendency for increased risk in female patients in the third tertile compared to the second tertile [HR 2.14 (95% CI 0.97–4.73), *p* = 0.058]. Moreover, in female patients the [first and the third tertile of log- neurotensin were associated with all-cause death 1s vs. 2nd tertile: HR 3.03 (95% CI 1.21–7.63), *p* = 0.018; 3rd vs. 2nd tertile: HR 3.01 (95% CI 1.22–7.44), *p* = 0.016].

**Conclusion:**

In female patients with CAD undergoing PCI, neurotensin has a U-shaped relationship with adverse outcomes. These data suggest a sex specific association between neurotensin and long-term adverse events after PCI.

## Introduction

Coronary artery disease (CAD) remains accountable for the majority of morbidity and mortality despite substantial advances in prevention, diagnosis and treatment (1). Significant efforts are undertaken to detect biomarkers reflecting an altered metabolic profile in order to enhance risk prediction and prognosis in patients with suspected or overt CAD.

Neurotensin is a small peptide that has been first extracted from bovine hypothalamus in the 1970s, and has subsequently been identified as a neurotransmitter of the central nervous system and as neuromodulator released from endocrine-like N-cells in the intestine (2, 3). Neurotensin is primarily secreted in response to fat ingestion and affects bowl mobility, pancreatic and biliary secretion and facilitation of fatty-acid translocation (4). Also, neurotensin knock-out mice are less likely to develop diet-induced obesity, insulin resistance and hepatic steatosis (5). Moreover, neurotensin has effects on the cardiovascular system by regulating heart rate, myocardial contractility and vascular tone (6). The effects of neurotensin are mediated by three receptors: The G-protein coupled neurotensin receptor type 1 (NTSR1) and 2 (NTSR2), and the non-G-protein coupled NTSR3, also known as sortilin-1 (7). Interestingly, in genome-wide association studies variants of NTSR3 have been associated with the incidence of CAD in humans (8).

Proneurotensin is the precursor of neurotensin and is released in equimolar amounts (9). Recent reports from population-based investigations associated fasting levels of proneurotensin with the incidence of diabetes, cardiovascular disease and mortality. Of note, these associations appear to be more pronounced in women compared to men (10–12).

Best to our knowledge, no data on the association of neurotensin with cardiovascular outcomes in patients with overt CAD undergoing percutaneous coronary intervention (PCI) have been reported. Therefore, we investigated the association of neurotensin on long-term adverse cardiovascular outcomes in patients presenting with acute coronary syndrome (ACS) or chronic coronary syndrome (CCS) undergoing PCI.

## Methods

### Patient Population

The Wilhelminenhospital Monitoring of Antiplatelet Activity (WILMAA) registry is a prospective single centre observational study. Consecutive patients undergoing PCI for ACS or CCS between May 2009 and December 2010 were included. Patients with platelet count below 100,000/ml, patients receiving any other antithrombotic agent than aspirin and clopidogrel and patients, who deceased during the initial hospital stay, were excluded from the present analysis.

Clopidogrel-naive patients received a 300 or 600 mg loading dose. Patients on chronic clopidogrel therapy with 75 mg clopidogrel of at least seven consecutive days did not receive an additional loading dose. A detailed depiction of the registry has been described previously (13, 14).

Summarized, diagnosis of ACS or CCS was established according to the guidelines effective at that time. ACS patients presented either with persistent ST-segment elevation myocardial infarction (STEMI) or non ST-elevation myocardial infarction (NSTEMI). Criteria for STEMI were biomarker evidence of MI with ST-segment elevation of 1 mm or more in two or more contiguous leads, while NSTEMI patients required elevated troponin I, troponin T or creatine-kinase MB (CK-MB) levels and/or ST-segment depression of ≥1 mm for diagnosis. CCS was defined according to positive ischemia testing (treadmill examination, dobutamine stress echocardiography or single-photon-emission computed tomography). All patients received clopidogrel and aspirin (acetylsalicylic acid) as dual antithrombotic therapy regimen. Implantation of drug eluting stents (DES) or bare metal stents (BMS), the use of peri-procedural anticoagulant regimen and further secondary-prevention medication were under the discretion of the treating interventionist. Laboratory results, clinical characteristics, cardiovascular risk factors, comorbidities, procedural details, and medication at hospital discharge were registered for all patients.

Our study complies with the Declaration of Helsinki of 1975, was approved by the local ethics committee (EK 09-0125-VK_NZ) and informed consent has been obtained from all subjects. The authors had full access to the data and take responsibility for its integrity.

### Determination of Neurotensin

Six to 24 h (h) after PCI (i.e. the next morning after PCI) venous blood samples were collected in fasting state via venepuncture of the antecubital vein into ethylenediaminetetraacetic acid tubes (Greiner BioOne, Kremsmünster, Austria). Neurotensin levels were measured using the Neurotensin (Human, Rat, Mouse) enzyme immunoassay kit (Phoenix Pharmaceuticals, Inc. CA, USA, detection range: 0 and 100 ng/ml) according to the manuals provided by the manufacturer.

### Clinical Endpoints

As primary endpoint, a composite of major adverse cardiovascular events (MACE) including all-cause death, non-fatal myocardial infarction (MI) and non-fatal ischemic stroke during seven years of follow-up was investigated. As secondary endpoint, we investigated all-cause death. Data were censored at time of adverse event or at end of follow-up. Mortality data for all patients were obtained from the Statistics Austria Institute. The Statistics Austria Institute is an independent and non-profitmaking federal institution under public law, which supports scientific services. Data on recurrent myocardial infarction or ischemic stroke was obtained by outpatient visits, telephone interviews or using the common Vienna regional hospital database system.

### Statistics

Since this registry was the first investigation relating neurotensin with MACE following PCI at the time, when the study was designed, no meaningful sample size calculation was possible. The study is observational and exploratory in nature.

All continuous variables are expressed as median [interquartile range (IQR)]. Categorical variables are given as number (%). Continuous variables were compared by Mann-Whitney *U*-test or Kruskal-Wallis *H*-test, as appropriate; χ^2^-tests were performed for comparison of categorical variables. General linear regression analysis with neurotensin as the dependent variable was performed using the augmented backward elimination algorithm (15). As first step, all potential prognostic variables were included into a step-wise backward elimination model using a likelihood-ratio test with a significance level of α > 0.2 for exclusion. In a second step, all primarily excluded variables were re-entered separately and kept in the model in case of a change-in-estimate of >5% in order to identify relevant confounders.

Receiver-operating characteristic (ROC) curve analysis was used to determine the ability of neurotensin to distinguish between patients without and with the endpoint of interest. Survival curves were calculated using the Kaplan-Meier method and compared using the log-rank test. Cox regression analysis was performed to estimate effect size and to allow for multivariable adjustment. Multivariable Cox proportional hazard models were applied according to the augmented backward elimination algorithm as described above (15). Partial residuals were used to assess the proportional hazard assumption and restricted cubic spline analysis (using 3 splines) was performed to assess the relationship of log-transformed neurotensin with our primary and secondary endpoint. All variables included in the final Cox regression models fulfilled the proportional hazard assumption. For multivariable analyses neurotensin was logarithmically transformed (log base 2) due to non-normal distribution. Following variables were included into the primary models (general linear regression and Cox regression analysis): neurotensin [dichotomized as tertiles due to non-linear associations with the primary and secondary endpoint in cubic spline analysis (Supplementary Figure 1)], age, sex, indication for PCI (ACS or CCS), arterial hypertension, type 2 diabetes mellitus (T2DM), tobacco abuse, prior myocardial infarction, prior ischemic stroke/transient ischemic attack, peripheral artery disease, heart failure (HF), atrial fibrillation, eGFR (according to CKD-EPI), peak troponin I, C-reactive protein (CRP), serum glucose, LDL-C, HDL-C, affected coronary vessels, stent length, the use of drug eluting stents, and discharge medication. In order to provide insight into differences in the impact of neurotensin on early and late MACE rates we performed additional analyses with a landmark set at 1 year.

All statistical tests were 2-tailed, and a *p*-value < 0.05 was required for statistical significance. All statistical analyses and figures were performed with R 4.1.3 (Vienna, Austria).

## Results

In total, 452 patients were eligible for analysis. Median age was 65 (55–75) years, and 144 (31.9%) were female. ACS was diagnosed in 268 (59.3%) patients, while CCS was present in 184 (40.7%). Clinical, laboratory, and procedural characteristics of the overall study population and stratified for male and female patients are presented in Table 1.

**Table 1:** Baseline characteristics.

	**All patients**	**Male patients**	**Female patients**	***P*** **-value**
	***N*** **= 452**	***N*** **= 308**	***N*** **= 144**	
Age, years	65.0 (55.0; 74.0)	61.0 (52.0; 70.0)	70.0 (62.0; 80.0)	<0.001
Neurotensin, ng/ml	0.30 (0.21; 0.43)	0.31 (0.21; 0.43)	0.30 (0.21; 0.40)	0.560
Indication for PCI				0.397
CCS	184 (40.7%)	130 (42.2%)	54 (37.5%)	
ACS	268 (59.3%)	178 (57.8%)	90 (62.5%)	
Heart rate, bpm	73 (62; 80)	72 (62; 79)	73.0 (62; 80)	0.550
Systolic blood pressure, mmHg	140 (125; 150)	140 (120; 151)	140 (125; 150)	0.938
Diastolic blood pressure, mmHg	80 (70; 90)	80 (75; 90)	80 (70; 90)	0.019
Body mass index, kg/m^2^	27.5 (25.3; 30.9)	27.7 (25.5; 30.5)	27.3 (24.4; 31.7)	0.833
Cardiogenic shock, No. (%)	11 (2.4%)	6 (1.9%)	5 (3.4%)	0.509
Hemoglobin, g/dl	13.5 (12.4; 14.5)	13.9 (13.0; 14.7)	12.5 (11.5; 13.5)	<0.001
Leukocyte count, G/l	9.1 (7.6; 10.5)	9.3 (7.8; 10.7)	8.7 (7.3; 9.8)	0.021
Platelet count, G/l	223 (185; 263)	217 (180; 254)	236 (197; 285)	0.001
Reticulated platelet count, G/l	7.65 (5.67; 10.3)	7.66 (5.61; 9.99)	7.63 (5.73; 10.5)	0.629
Creatinine, mg/dl	0.90 (0.79; 1.10)	0.95 (0.83; 1.11)	0.80 (0.69; 0.95)	<0.001
eGFR, ml/min/1.73 m2	82.0 (64.4; 96.6)	83.9 (67.8; 97.8)	74.5 (58.0; 94.1)	0.001
Serum glucose at admission, mg/dl	119 (100; 136)	117 (99.9; 133)	123 (100; 146)	0.098
Troponin I, ng/l	2.08 (0.14; 18.4)	2.12 (0.13; 18.4)	1.94 (0.20; 18.4)	0.526
C-reactive protein, mg/l	6.86 (2.22; 18.4)	6.24 (1.95; 18.4)	7.74 (2.70; 18.4)	0.440
VASP-P, PRI	63.6 (48.3; 75.0)	64.6 (49.5; 76.0)	61.4 (43.4; 72.2)	0.048
MEA ADP, AU	34.8 (24.0; 41.2)	34.8 (25.0; 42.0)	34.8 (21.0; 40.2)	0.787
Heart failure, No. (%)	52 (11.5%)	31 (10.1%)	21 (14.6%)	0.213
Atrial fibrillation, No. (%)	36 (7.9%)	22 (7.1%)	14 (9.7%)	0.449
Type 2 diabetes mellitus, No. (%)	131 (29.0%)	83 (26.9%)	48 (33.3%)	0.200
Arterial hypertension, No. (%)	384 (85.0%)	255 (82.8%)	129 (89.6%)	0.082
Hyperlipidemia, No. (%)	356 (78.8%)	247 (80.2%)	109 (75.7%)	0.334
Prior myocardial infarction, No. (%)	121 (26.8%)	85 (27.6%)	36 (25.0%)	0.640
Prior revascularization, No. (%)	120 (26.5%)	78 (25.3%)	42 (29.2%)	0.455
Prior stroke or TIA, No. (%)	30 (6.6%)	22 (7.1%)	8 (5.5%)	0.668
Peripheral artery disease, No. (%)	44 (9.7%)	29 (9.4%)	15 (10.4%)	0.870
Access site, No. (%)				0.309
Femoral	435 (96.2%)	294 (95.5%)	141 (97.9%)	
Radial	17 (3.7%)	14 (4.5%)	3 (2.1%)	
Use of DES, No. (%)	303 (67.0%)	212 (68.8%)	91 (63.2%)	0.280
Affected coronary vessels, No. (%)	3 (2; 3)	3 (2; 3)	2 (2; 3)	0.021
Total stent length, mm	24 (18; 36)	24 (18; 38)	24 (18; 32)	0.180
Beta blocker, No. (%)	357 (79.0%)	247 (80.2%)	110 (76.4%)	0.423
ACE-I or ARB, No. (%)	306 (67.7%)	212 (68.8%)	94 (65.3%)	0.519
Statin, No. (%)	416 (92.0%)	282 (91.6%)	134 (93.1%)	0.718
Oral antidiabetic medication, No. (%)	91 (20.1%)	64 (20.8%)	27 (18.8%)	0.707
Insulin, No. (%)	27 (5.9%)	15 (4.9%)	12 (8.3%)	0.217

*Data are depicted as median (IQR) or as absolute numbers (%). ACE-I, angiotensin-converting enzyme inhibitor; ACS, acute coronary syndrome; ADP, adenosine diphosphate; ARB, angiotensin-receptor blocker; CCS, chronic coronary syndrome; DES, drug-eluting stent; MEA, multiple-electrode aggregometry; PCI, percutaneous coronary intervention; TIA, transient ischemic attack*.

**Figure 1:**
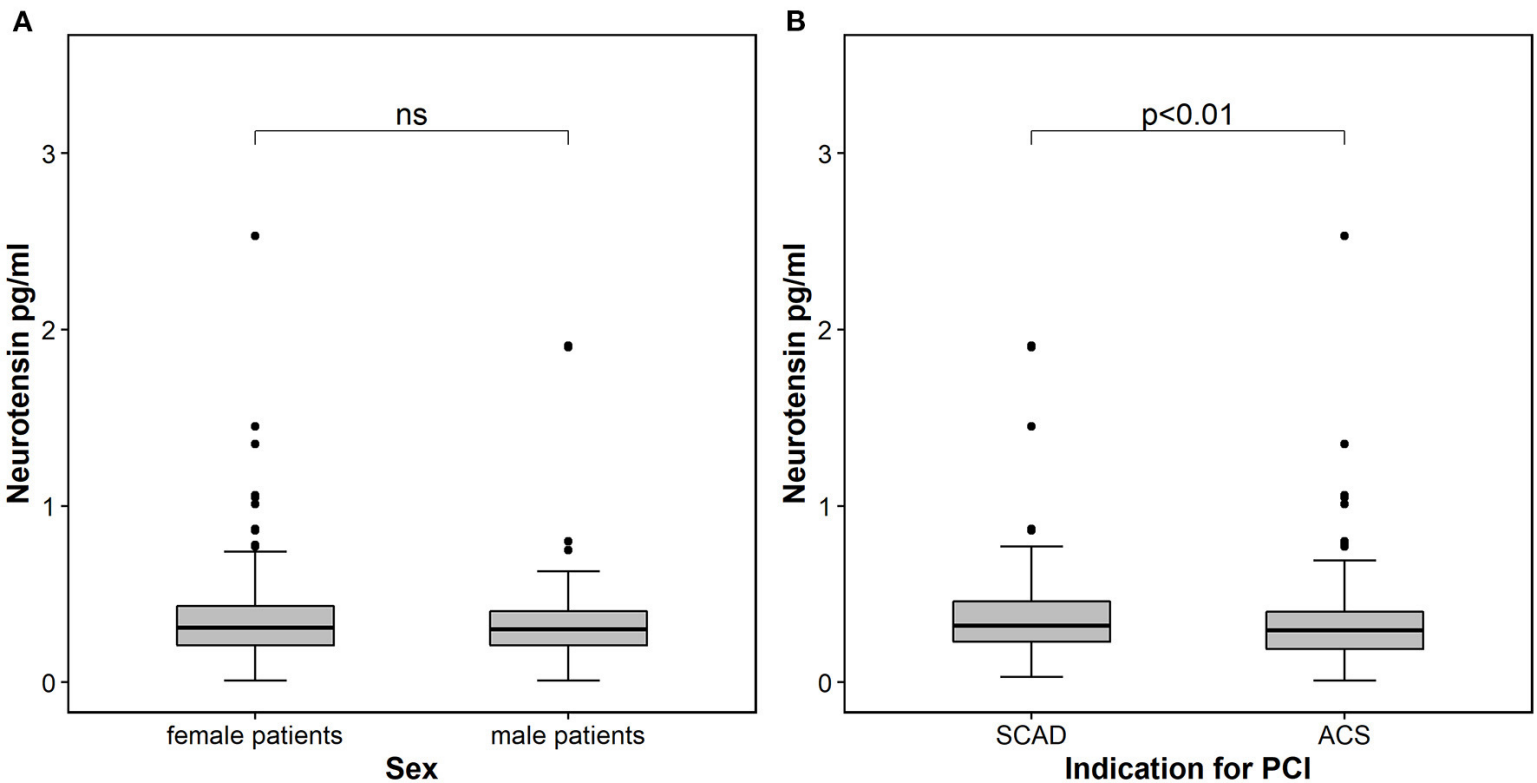
Neurotensin in patients stratified according to sex **(A)** and according to indication for percutaneous coronary intervention **(B)**. The boundaries of the box show the lower and upper quartile of data and the line inside the box represents the median. Whiskers are drawn from the edge of the box to the highest and lowest values that are outside the box but within 1.5 times the box length. The outliers are presented as dots. ACS, acute coronary syndrome; SCAD, stable coronary artery disease; PCI, percutaneous coronary intervention.

Neurotensin levels after PCI did not differ between male and female patients (*p* = 0.560, Table 1; Figure 1), however patients with CCS had significantly higher levels compared to patients with ACS [0.32 ng/ml (IQR 0.23–0.46) vs. 0.29 ng/ml (IQR 0.19–0.40); *p* < 0.01; Figure 1]. In multivariable linear regression analysis HF (β = 0.496; *p* = 0.001) was positively associated, whereas clinical presentation for ACS (β = −0.238; *p* = 0.015) and troponin I (β = −0.002; *p* = 0.005) were negatively associated with neurotensin (Table 2).

**Table 2:** Determinants of neurotensin in a general linear regression model using the augmented backward elimination algorithm.

**Parameters of the final model**	**β coefficient**	***P*** **-value**
Body mass index	−0.014	0.158
VASP-P	−0.003	0.145
MEA, ADP	0.004	0.172
Sex	−0.133	0.201
Indication for PCI	−0.238	0.015
Tobacco abuse	−0.191	0.056
Heart failure	0.496	0.001
Troponin I	−0.002	0.005

*ADP, adenosine diphosphate; MEA, multiple electrode aggregometry; PCI, percutaneous coronary intervention. Neurotensin was logarithmically transformed for this analysis. Adjustment was performed for risk factors, co-morbidities, procedural details and discharge therapy. Variables included in the model prior to elimination are listed in the statistical methods section*.

### Outcome Analysis

**Figure 2:**
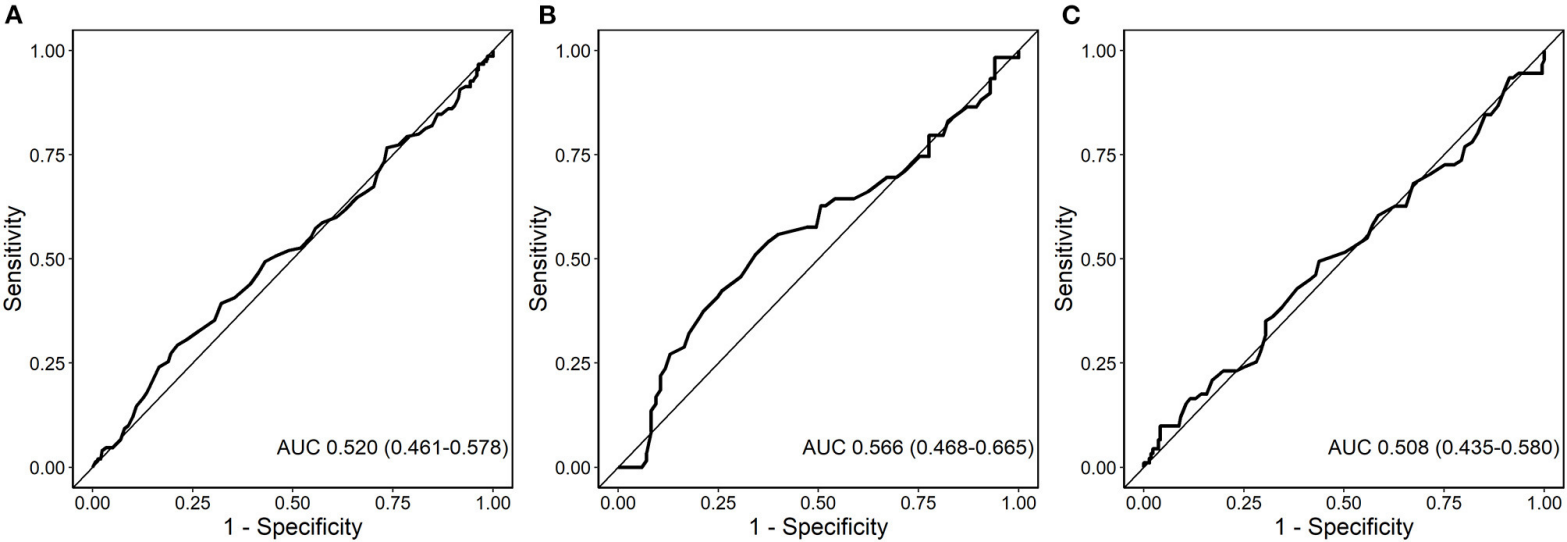
Receiver-operating characteristics (ROC) curve for the analysis of the predictive value of neurotensin with major adverse cardiovascular events (MACE) for all patients **(A)**, female patients **(B)**, and male patients **(C)**.

**Figure 3:**
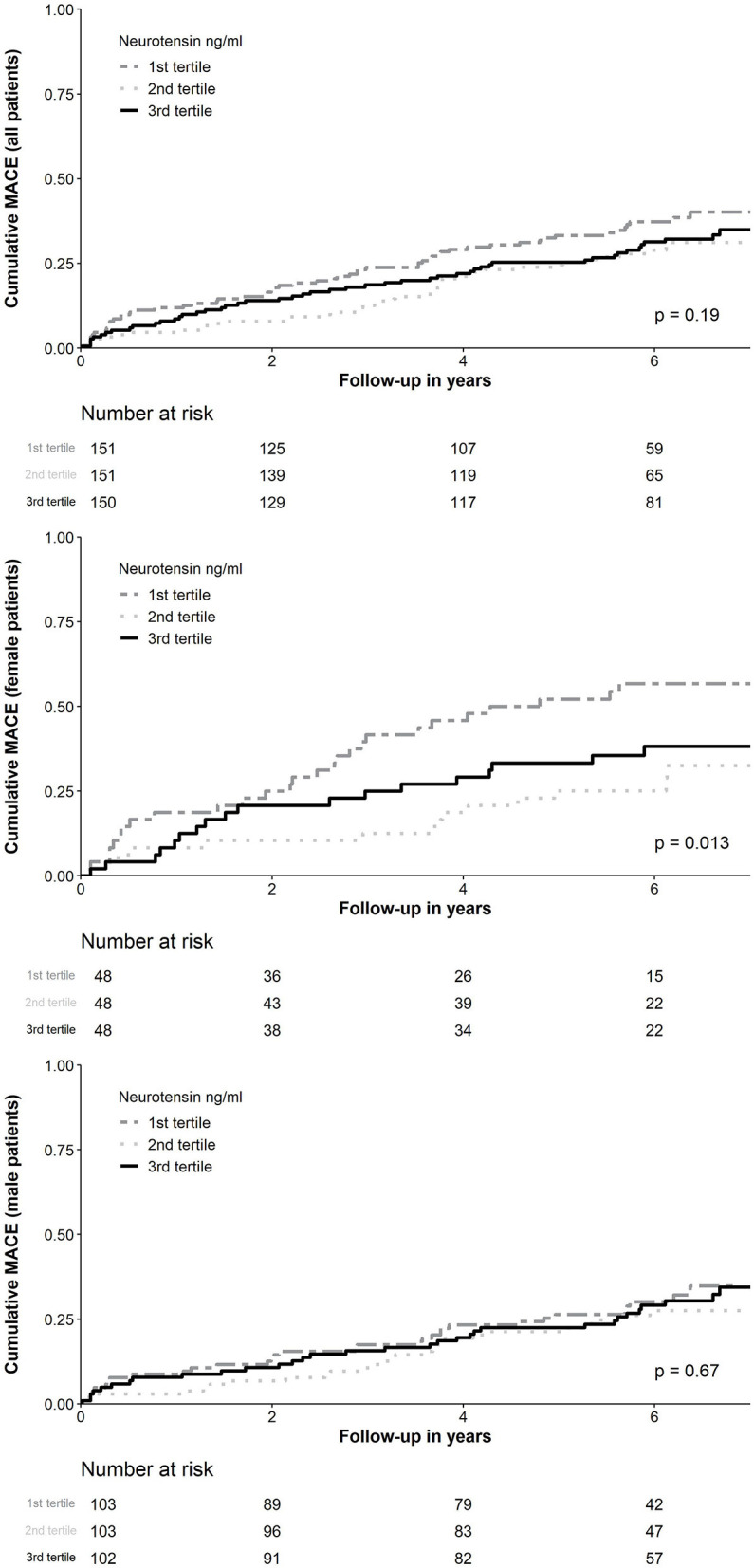
Kaplan-Meier curve analysis for neurotensin according to tertiles for all patients and stratified for female and male patients. MACE, Major adverse cardiovascular events.

MACE occurred in 150 (33.2%) patients, including 89 (19.0%) patients with all-cause death, 47 (10.2%) with non-fatal MI and 17 (3.8%) with non-fatal stroke. In ROC analysis neurotensin did not discriminate between patients with and without MACE for all patients (*p* = 0.500), as well as stratified for male (*p* = 0.820) and female (*p* = 0.180) patients, respectively (Figure 2). Univariable Cox regression analysis including restricted cubic splines demonstrated a non-linear U-shaped association of log-transformed neurotensin with the primary and secondary endpoint (Supplementary Figure 1). Therefore, we dichotomized our cohort according to tertiles of log-transformed neurotensin. In Kaplan-Meier analysis including the total cohort log-transformed neurotensin tertiles were not associated with MACE (*p* > 0.05, Figure 3). Similarly, in multivariable Cox regression analysis log-transformed neurotensin tertiles were not associated with MACE or with all-cause death (all *p* > 0.05). In order to evaluate the impact of BMI on our Cox regression model as known mediator of neurotensin, we performed a step-wise approach using 3 models, as demonstrated in Table 4. We could not detect a relevant impact of BMI.

**Table 3:** Predictors of long-term major adverse cardiovascular events for all patients using the augmented backward elimination algorithm.

**Parameters of the final Cox regression model^a^**	**HR (95%CI)**	***P*** **-value**
**Neurotensin, log transformed**		
1^st^ tertile	1.438 (0.950–2.176)	0.085
2^nd^ tertile (Ref.)	Ref.	
3^rd^ tertile	1.209 (0.797–1.833)	0.370
Age, years	1.030 (1.012–1.049)	0.001
Body-mass index, kg/m2	0.957 (0.920–0.996)	0.034
ACS	1.317 (0.926–1.874)	0.125
Heart failure	1.615 (1.010–2.582)	0.045
Diabetes mellitus	1.562 (1.092–2.232)	0.014
Prior myocardial infarction	1.354 (0.945–1.940)	0.097
Peripheral artery disease	2.179 (1.379–3.445)	0.001
Troponin I, ng/l	1.003 (1.001–1.005)	0.002
eGFR, ml/min	0.988 (0.979–0.997)	0.015
ACE-I or ARB	0.563 (0.398–0.795)	0.001

*ACE-I, angiotensin-converting enzyme inhibitor; ARB, angiotensin-receptor blocker; HR, hazard ratio; CI, confidence interval, eGFR, estimated glomerular filtration rate*.

a *Adjustment was performed for risk factors, co-morbidities, procedural details and discharge therapy. Variables included in the model prior to elimination are listed in the statistical methods section*.

**Table 4:** Cox regression models for log-transformed neurotensin stratified in tertiles and long-term major adverse cardiovascular events using the variables of the final model derived from the augmented backwards regression (see Table 3).

**Parameter**	**HR (95% CI)**	***P-value***
**Model 1** ^a^
**Neurotensin, log transformed**		
1^st^ tertile	1.594 (1.072–2.367)	0.021
2^nd^ tertile	Ref.	
3^rd^ tertile	1.144 (0.760–1.721)	0.518
**Model 2** ^b^		
**Neurotensin, log transformed**		
1^st^ tertile	1.463 (0.965–2.219)	0.073
2^nd^ tertil	Ref.	
3^rd^ tertile	1.232 (0.810–1.875)	0.329
**Model 3** ^c^		
**Neurotensin, log transformed**		
1^st^ tertile	1.453 (0.958–2.203)	0.078
2^nd^ tertile	Ref.	
3^rd^ tertile	1.224 (0.806–1.858)	0.342

*HR, hazard ratio; CI, confidence interval*.

a *Model 1, adjusted for age and sex*.

b *Model 2, Model 1 ± adjusted for the variables of the final augmented regression analysis excluding BMI*.

c *Model 3, Model 2 ± adjusted for BMI*.

Similarly, examining risk across log-transformed neurotensin tertiles in male patients did not demonstrate a significant association with MACE and all-cause death in Kaplan-Meier and multivariable Cox regression analysis (all *p* > 0.05, Figure 3; Table 5) However, in female patients log-transformed neurotensin stratified into tertiles was associated with MACE in Kaplan-Meier analysis (log-rank *p* = 0.013, Figure 3). Also, after multivariable adjustment female patients in the first tertile had a significantly increased risk for MACE compared to female patients in the second tertile [HR 3.84 (95% CI 1.71–8.60), *p* = 0.001]. Also, there was tendency for increased risk in female patients in the third tertile compared to the second tertile [HR 2.14 (95% CI 0.97–4.73), *p* = 0.058], as shown in Table 5. Moreover, in female patients the first and the third tertile of log-transformed neurotensin were associated with all-cause death as compared to the second tertile [1st vs. 2nd tertile: HR 3.03 (95% CI 1.21–7.63), *p* = 0.018; 3rd vs. 2nd tertile: HR 3.01 (95% CI 1.22–7.44), *p* = 0.016].

**Table 5:** Multivariable Cox regression model for neurotensin and long-term major adverse cardiovascular events in male and female patients using the augmented backward elimination algorithm.

**Parameter**	**HR (95%CI)**	***P*** **-value**
**Male patients**		
**Neurotensin, log transformed**		
1^st^ tertile	1.487 (0.866–2.554)	0.150
2^nd^ tertile (Ref.)	Ref.	
3^rd^ tertile	1.228 (0.726–2.075)	0.442
Age, years	1.020 (0.995–1.045)	0.102
Peripheral artery disease	2.766 (1.552–4.931)	0.001
Glucose, mg/dl	1.005 (1.002–1.008)	0.001
LDL-cholesterol, mg/dl	0.995 (0.989–1.001)	0.126
eGFR, ml/min	0.990 (0.977–1.003)	0.143
Troponin I, ng/l	1.003 (1.001–1.006)	0.011
Use of drug-eluting stents	1.485 (0.913–2.414)	0.110
Number of affected coronary vessel	1.698 (1.275–2.261)	0.001
Total stent length, mm	0.979 (0.965–0.994)	0.006
ACE-I or ARB	0.555 (0.351–0.879)	0.012
**Female patients**		
**Neurotensin, log transformed**		
1^st^ tertile	3.835 (1.710–8.596)	0.001
2^nd^ tertile (Ref.)	Ref.	
3^rd^ tertile	2.144 (0.972–4.729)	0.058
Age, years	1.063 (1.024–1.103)	0.001
Body-mass-index, kg/m^2^	0.974 (0.916–1.035)	0.395
Smoking	2.888 (1.456–5.730)	0.002
Arterial hypertension	0.324 (0.136–0.774)	0.011
Heart failure	1.811 (0.874–3.753)	0.109
Prior myocardial infarction	2.661 (1.474–4.802)	0.001
Prior stroke or TIA	2.572 (0.861–7.687)	0.090
Troponin I, ng/l	1.004 (0.997–1.012)	0.203
eGFR, ml/min	0.980 (0.964–0.996)	0.016
Glucose, mg/dl	0.997 (0.993–1.002)	0.314
LDL-cholesterol, mg/dl	1.014 (1.005–1.024)	0.002
HDL-cholesterol, mg/dl	0.984 (0.961–1.008)	0.203
Use of drug-eluting stents	0.430 (0.242–0.766)	0.004
ACE-I or ARB	0.470 (0.253–0.874)	0.017
Insulin therapy	3.732 (1.308–10.649)	0.013

*HR, hazard ratio; CI, confidence interval; ACE-I, angiotensin-converting enzyme inhibitor; ARB, angiotensin-receptor blocker; eGFR, estimated glomerular filtration rate; HDL, high-density lipoprotein; TIA, transient ischemic attack. Adjustment was performed for risk factors, co-morbidities, procedural details and discharge therapy. Variables included in the model prior to elimination are listed in the statistical methods section*.

### Landmark Analysis

Analyzing the impact of log-transformed neurotensin on short-term MACE censored after 1-year of follow-up, we could not detect a significant association in the overall cohort (log-rank *p* = 0.077, Supplementary Figure 2). In a landmark analysis excluding all patients with MACE within the first 12 months of follow-up examining the risk across log-transformed neurotensin tertiles we found no association with MACE in the overall cohort and in male patients, respectively (both log-rank *p* > 0.05). However, we found a significant difference in female patients (log-rank *p* = 0.020), as depicted in Supplementary Figure 3.

## Discussion

The main finding of the present analysis is that neurotensin levels post PCI demonstrated a U-shaped relationship with long-term adverse outcomes in patients undergoing coronary stenting for ACS and CCS. Stratified into tertiles we could not detect a significant association of neurotensin levels after PCI with MACE or all-cause death in the overall cohort and in male patients. However, female patients in the first and third tertile of neurotensin post PCI were at increased risk for MACE and all-cause death. Finally, neurotensin post PCI was not associated with parameters of metabolic profile.

Previous observational investigations including participants without overt cardiovascular disease reported a positive linear association of proneurotensin, released in equimolar amounts with neurotensin, levels with future incidence of cardiovascular disease and mortality (10–12). In the population-based “Malmo Diet and Cancer Study” fasting proneurotensin was associated with the development of T2DM, cardiovascular disease and with total and cardiovascular mortality during a follow-up period of 13.2 years (10). Similar results have been reported from the “Framingham Heart Offspring” cohort and from the “Malmo Preventive Project” (11, 12). Of note, the associations of proneurotensin with adverse cardiovascular outcomes were more pronounced in female than in male patients, a finding confirmed by our results (10–12).

However, a pathophysiological link of neurotensin with atherosclerosis remains yet unknown: Although NTS*R3* has been associated with the formation of atherosclerotic lesions and the incidence of CAD and myocardial infarction, the effect seems to be independent of neurotensin, as NST*R3* acts as well as a receptor for several pro-atherogenic proteins (i.e., interferon-γ and interleukin-6) (8, 11, 16, 17). Also, NT*S1* has not been associated with atherosclerotic processes, and its cardiovascular effects are restricted to vascular tone, heart rate and myocardial contractility, whereas NT*S2* has no known cardiovascular effects (6). Already Melander et al. suggested proneurotensin to be a mere marker of underlying disease susceptibility, as proneurotensin predicted adverse events many years in advance (10–12). This assumption is supported by our findings demonstrating a U-shaped relationship of neurotensin with MACE and all-cause death in patients with overt cardiovascular disease as opposed to the former studies including predominantly healthy participants bringing into question a mechanistic role of neurotensin in atherosclerotic processes. Moreover, our results are confirmed by a recent study reporting a U-shaped association of proneurotensin with premature coronary artery disease (18).

Interestingly, in our cohort neurotensin levels post PCI were lower in patients with ACS as compared to patients with CCS and neurotensin was negatively associated with peak troponin levels in regression analysis. Previous investigations have demonstrated significant alterations of metabolic parameters such as lipoproteins and serum glucose levels in the acute setting of ACS, possibly also affecting neurotensin levels (19, 20).

Of note, we are the first to report a clinical association of neurotensin with HF, which is supported by several prior studies, as neurotensin exhibits a strong inotropic effect on myocardial cells (6, 21). Also, angiotensin-converting enzyme 2 (ACE2), cleaves C-terminal residues of several important vasoactive peptides including neurotensin (22). These and our data advocate a significant role of neurotensin in HF.

Although our results do not support a mechanistic function of neurotensin in advanced atherosclerotic disease, neurotensin drawn after coronary stenting might serve as a prognostic marker particularly in female patients undergoing PCI for ACS and CCS. More studies are needed to further investigate the pathophysiological role of neurotensin in early stages of atherosclerosis and HF and its value in risk prediction and as a potential therapeutic target.

## Limitations

The present investigation should be interpreted considering the following limitations: These results were derived from a single-centre patient population undergoing PCI. Therefore, our results cannot be extrapolated to patients with CAD treated medically and those who received surgical revascularization. Furthermore, neurotensin levels were measured at a single time point after stent implantation and we cannot rule out an impact of PCI on neurotensin levels. Also, in the present study all patients were treated with DAPT including aspirin and clopidogrel but not novel antiplatelet drugs. Due to small numbers of patients with subsequent non-fatal MI and non-fatal stroke no outcome analyses were performed. Finally, in the literature the measurement of proneurotensin was advocated due to *in vivo* and *in vitro* instability of neurotensin (9). However, the cited experiments included the exposure of neurotensin to dog ileum and to cell cultures including human colon adenocarcinoma cells and might therefore not apply to samples measured in human serum (23, 24).

## Conclusion

We demonstrated a U-shaped relationship of neurotensin post-PCI with adverse outcomes in patients undergoing coronary stenting for CCS or ACS. Neurotensin was not associated with outcomes in male patients, but might serve as a risk marker for subsequent adverse events in female patients with CAD.

## Data Availability Statement

The raw data supporting the conclusions of this article will be made available by the authors, without undue reservation.

## Ethics Statement

The studies involving human participants were reviewed and approved by Ethikkommission der Stadt Wien, Vienna, Austria. The patients/participants provided their written informed consent to participate in this study.

## Author Contributions

SF, KH, and AK-W designed the study and developed the study concept. SF, BV, MF, IT, FT, and MR were responsible for patient inclusion and executed the acquisition of samples. SF, ML, SB-P, and AK-W were involved in the laboratory processing and methods. MT and SF processed and analyzed the data. MT wrote the original draft and designed all figures and tables. SF, TW, KH, and AK-W reviewed and edited the manuscript. All authors contributed to the article and approved the submitted version.

## Funding

This work was supported by the Association for the Promotion of Research in Atherosclerosis, Thrombosis and Vascular Biology (ATVB), and by the Ludwig Boltzmann Foundation for Cardiovascular Research, Vienna, Austria.

## Conflict of Interest

AK-W was employed by the Gender Institute, Lapura Women's Health Resort, Gars am Kamp, Austria. The remaining authors declare that the research was conducted in the absence of any commercial or financial relationships that could be construed as a potential conflict of interest.

## Publisher's Note

All claims expressed in this article are solely those of the authors and do not necessarily represent those of their affiliated organizations, or those of the publisher, the editors and the reviewers. Any product that may be evaluated in this article, or claim that may be made by its manufacturer, is not guaranteed or endorsed by the publisher.

## Supplementary Material

Supplementary Figure 1 Restricted cubic spline analysis assessing the relationship of log-transformed neurotensin with MACE **(A)** and all-cause death **(B)**. MACE, Major adverse cardiovascular events.

Supplementary Figure 2 Kaplan-Meier curve analysis for neurotensin according to tertiles for all patients assessing 1-year MACE. MACE, Major adverse cardiovascular events.

Supplementary Figure 3 Kaplan-Meier curve analysis for neurotensin according to tertiles for all patients and stratified for female and male patients (landmark). MACE, Major adverse cardiovascular events.
