# Supplementary figures and images for: Neurotensin and Adverse Cardiovascular Outcomes in Patients Undergoing Percutaneous Coronary Intervention
Source: Front Cardiovasc Med. 2022 Mar 8;9:782602. doi: 10.3389/fcvm.2022.782602 (PMC8957262; doi:10.3389/fcvm.2022.782602)

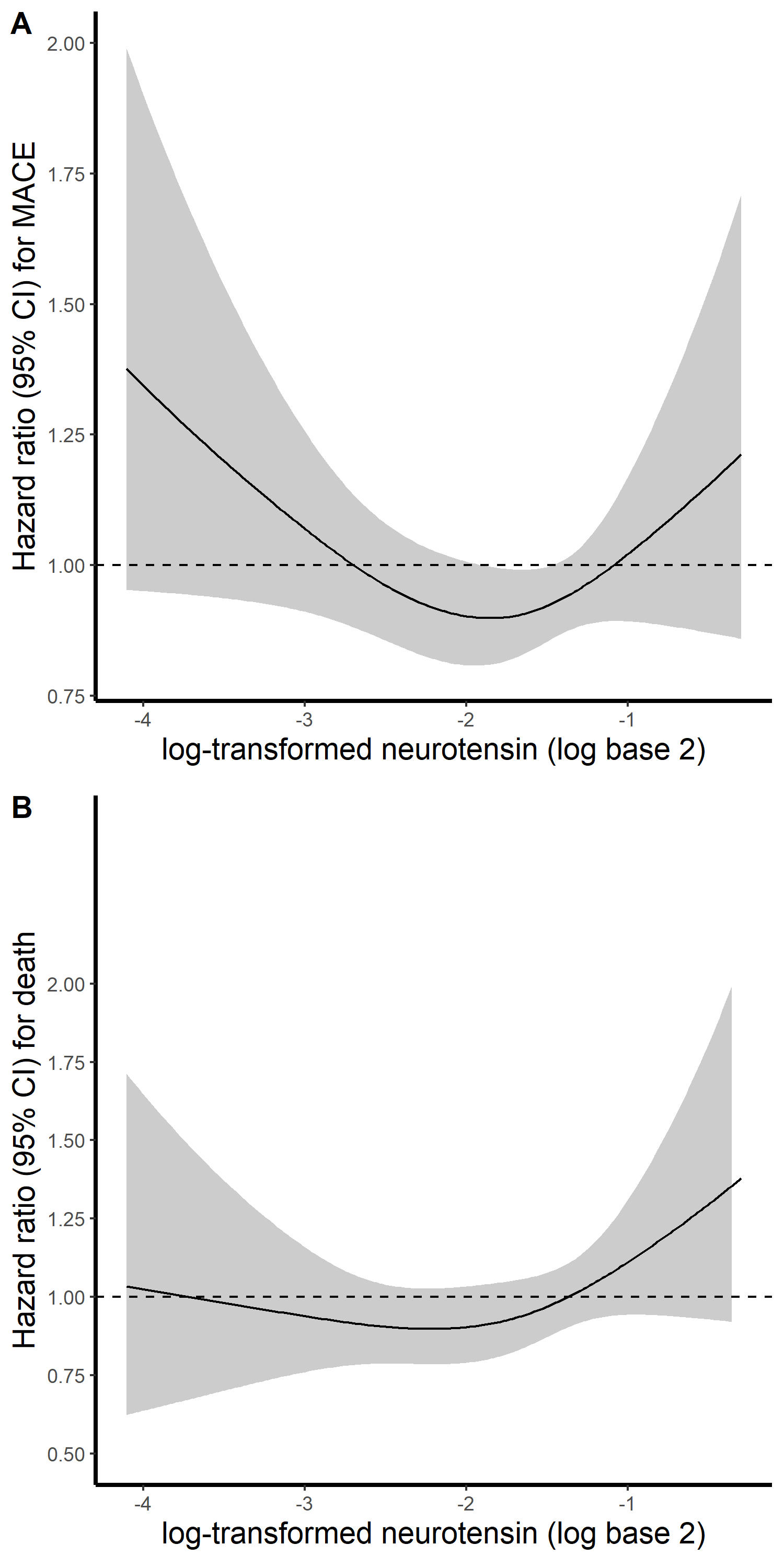

Supplement: Supplementary Figure 1 — Restricted cubic spline analysis assessing the relationship of log-transformed neurotensin with MACE (A) and all-cause death (B). MACE, Major adverse cardiovascular events. [file Image_1.TIFF]

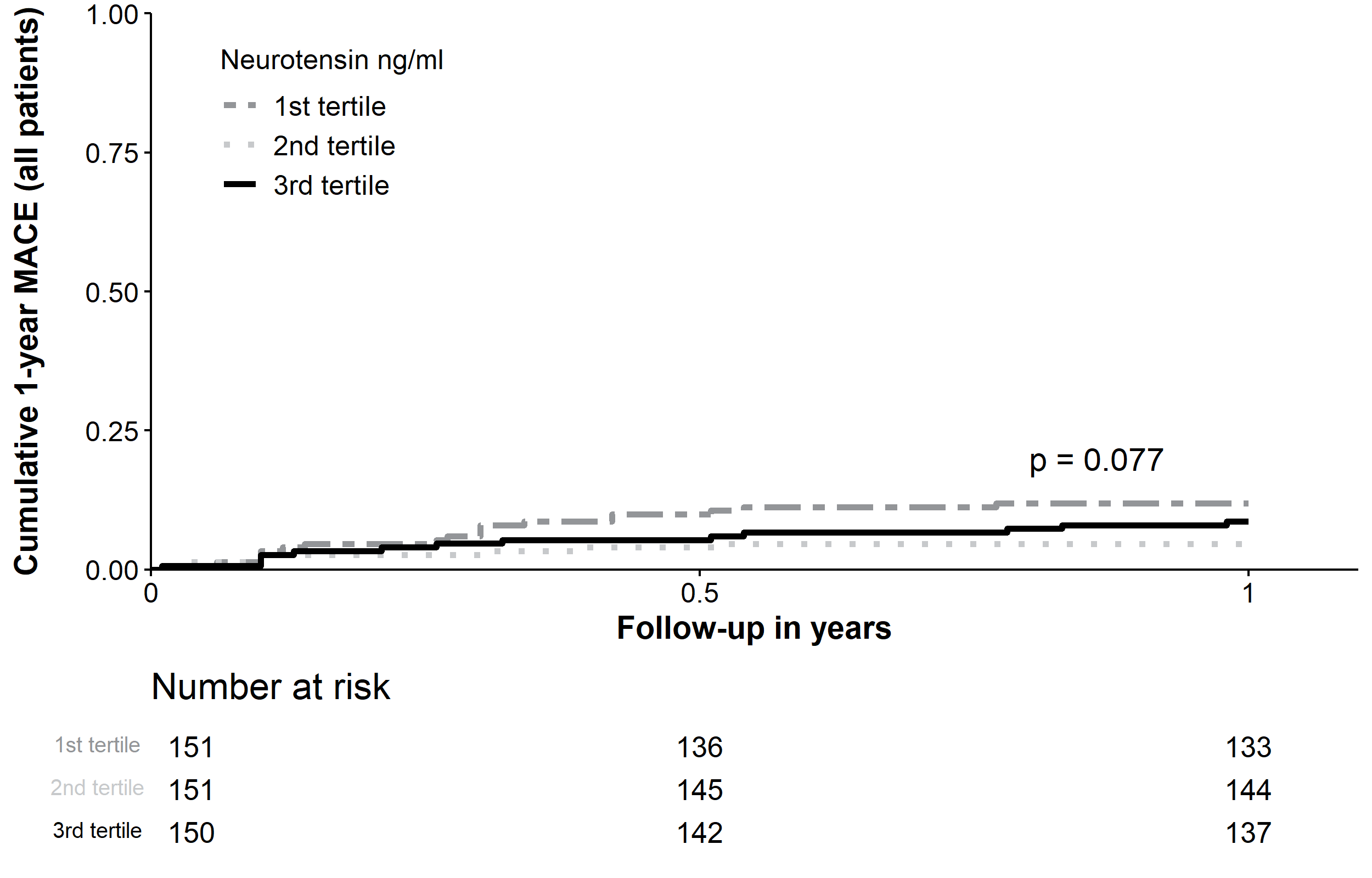

Supplement: Supplementary Figure 2 — Kaplan-Meier curve analysis for neurotensin according to tertiles for all patients assessing 1-year MACE. MACE, Major adverse cardiovascular events. [file Image_2.TIFF]

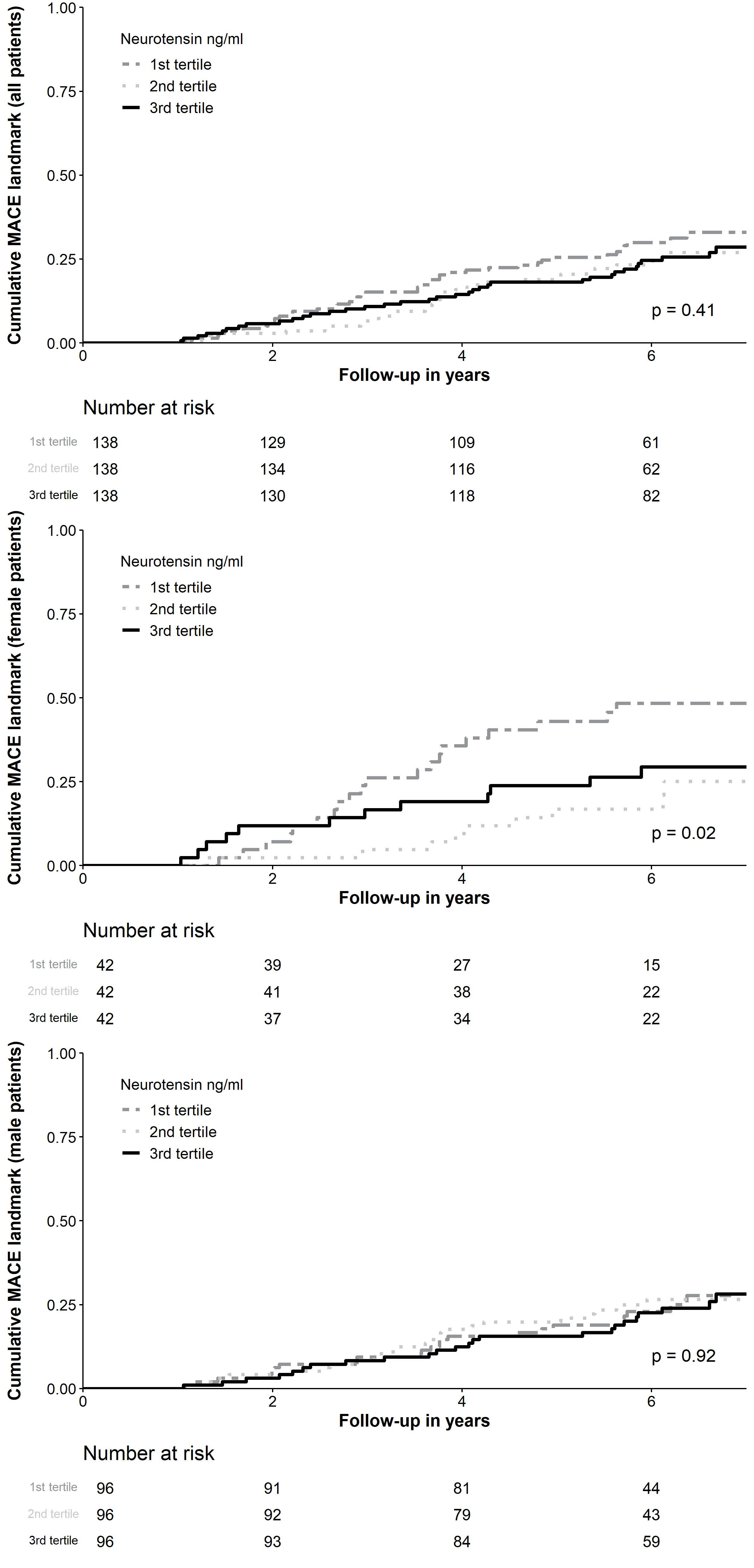

Supplement: Supplementary Figure 3 — Kaplan-Meier curve analysis for neurotensin according to tertiles for all patients and stratified for female and male patients (landmark). MACE, Major adverse cardiovascular events. [file Image_3.TIF]
